# "The Hospital" Medical Book Supplement.—No. XVI

**Published:** 1909-03-20

**Authors:** 


### "THE HOSPITAL" MEDICAL BOOK SUPPLEMENT.—No. XVI.

CONTENTS.

**American Post-graduate Archives—**
Contributions to the Science of Medicine and Surgery ... ... 645
**Tropical Medicine—**
Third Report of the Wellcome Research Laboratories at the Gordon Memorial College, Khartoum ... ... ... ... 646
**Therapeutics—**
A Manual of Medical Treatment, or Clinical Therapeutics ... 646
Physical Methods in the Treatment of Heart Disease ... ... 646
**Zoology—**
The Origin of Vertebrates ... ... ... ... ... ... 647
**Surgery—**
Injuries and Diseases of the Knee-joint ... ... ... ... 647
Seven Hundred Surgical Suggestions ... ... ... ... 647
**Miscellaneous Items—**
The Local Government Annual and Departmental Decisions by the Local Government Board ... ... ... ... ... 648
The Food Inspector's Handbook ... ... ... ... 643
An Introduction to the Science of Radio-Activity ... ... 648
The Body at Work ... ... ... ... ... ... 648

### AMERICAN POST-GRADUATE ARCHIVES.

CONTRIBUTIONS TO THE SCIENCE OF MEDICINE AND SURGERY BY THE FACULTY IN CELEBRATION OF THE TWENTY-FIFTH ANNIVERSARY (1882-1907) OF THE FOUNDING OF THE NEW YORK POST-GRADUATE MEDICAL SCHOOL AND HOSPITAL, 1908. Edited by HENRY T. BROOKS, M.D. New York.

THIS celebration volume consists of forty-eight papers upon various medical and surgical subjects by different authors, extending over nearly 500 pages, and illustrated by some first-class plates and figures. Many of the papers are of great interest. Dr. McKernon records a case of mastoiditis complicated by purulent meningitis, encephalitis, phlebitis of the sigmoid sinus, jugular bulb, and internal jugular vein, yet ending in recovery after operation. It is good to hear that even so extensive and grave an infection from the ear may get well. The fact that the micro-organism was the diplococcus intracellularis meningitidis probably had something to do with the recovery. Dr. Quintard writes upon hepato-intestinal toxæmia; he does not give the arguments upon which he bases his opinion that such a malady exists; he assumes that it does, and devotes himself solely to the clinical and therapeutical aspects of the illnesses of which he presumes hepato-intestinal toxæmia is the cause; there is little or no mention of the lactic acid bacillary treatment of the condition so much advocated lately, but a good many other therapeutic hints and suggestions are given. Dr. de Garmo gives a valuable account of wounds of the bladder occurring in the course of radical cures of herniæ, and publishes twenty personal cases besides referring to those in the literature. Dr. Emmet deals in a short paper with the use of uterine dilatation, the essence of which is that hysterectomy might sometimes be avoided if efficient dilatation of the uterine body were resorted to in suitable cases more often than it is. Dr. Weber writes upon the significance of gastro-intestinal toxins in the development and maintenance of pernicious anæmia; he gives four illustrative cases, but he does little more than re-state the well-known view that gastro-intestinal toxins do cause pernicious anæmia; he adduces no real proof in its favour. Dr. Chapin discusses "Biology as the Basic Principle in Infant-feeding," this being his thirteenth article on the subject; his points are interesting, and should appeal to those who have many infants under their charge. Dr. Beck advocates partial thyroidectomy combined with Röntgen treatment in Graves' disease; his paper is illustrated, and his conclusions are: (1) that slight thyroid enlargement in Graves' disease should be treated by the Röntgen-diaphragm method at short intervals; (2) that large Graves' goitre should be treated by the combination method, namely, removal of the larger lobe under local anæsthesia and without the use of antiseptics of any kind; the other portion to be irradiated as soon as the reaction from the operation is over; (3) that in advanced cases of Graves' disease, where alarming symptoms forbid immediate operative measures, Röntgen treatment should precede the operation until improvement has occurred. Dr. Cole lays stress upon the importance of local applications possessing nutritive value in causing chronic ulcers of any sort to heal; amongst the substances he uses are cod-liver oil, peptone, creatine and meat extractives, albumen, gelatine, ether-extracted fat, and alcohol and water. Dr. Lloyd speaks of the surgical treatment of "Unresolved pneumonia." His conclusion seems to be that many cases of so-called unresolved pneumonia are really undiagnosed empyemata, and that they should be operated upon as such. Dr. Bryant describes an operation termed mastoideo-tympanotomy, which he regards as the operation of election for persistent otorrhœa and acute mastoideo-tympanic osteitis. Dr. Taylor gives a short but interesting account of fresh-air work for surgical tuberculosis, with illustrations showing patients getting fresh-air in city surroundings, on tenement roofs, on the fire escape, through open windows, on porches, in the front yard, or in the city park; the paper demonstrates that good open-air measures may be devised and carried out in apparently hopeless circumstances. Dr. Peterson writes upon cicatricial stricture of the œsophagus, and concludes that: (1) forcible introduction of bougies, rapid instrumental divulsion, internal œsophagotomy with sharp-cutting instruments, are positively dangerous and should not be employed; (2) before attempting any dilatation of an œsophageal stricture a guide bougie or string-guide should be passed; (3) the treatment does not end with the operation. Dr. Kast gives a summary of investigations upon excessive gastric secretion, denies that there is any such thing as actual hyperchlorhydria in the sense that the juice itself becomes abnormally acid, and thinks that many gastric disorders now regarded as neuroses are really due to hypersecretion from excessive formation of hormones in the mucous membrane of the stomach and duodenum.

It is a pity that the volume has only a paper cover, and that it has no index beyond a meagre list of contents. It is obviously impossible, even in a lengthy review, to mention many of the papers here collected. A considerable number of them are of great importance and interest. All medical libraries should make a point of possessing this volume, and it is of interest to specialists and to general practitioners alike from all sorts of aspects, including those of the physician, the general surgeon, the ophthalmic surgeon, the genito-urinary surgeon, the aural surgeon, the gynæcologist and obstetrician, and the alienist. We think it constitutes an admirable anniversary publication, and we hope it may be repeated at a much shorter interval than twenty-five years. Might it not be a yearly issue on the lines of the Annual Reports, which are produced by the senior staffs of the great London Hospitals?

### TROPICAL MEDICINE.

Third Report of the Wellcome Research Laboratories at the Gordon Memorial College, Khartoum. Andrew Balfour, M.D., B.Sc., F.R.C.P., Edin., D.P.H.Camb., Director. (Published for Department of Education, Soudan Government, Khartoum, by Baillière, Tindall, and Cox. London, 1908. Pp. 450. 48 plates. 218 illustrations. Price, one guinea.)

Many very interesting papers are contained in the above report. In addition to the director (Dr. Balfour) and the staff of the laboratories numerous other gentlemen have written papers on a variety of subjects which enhance the value of the publication. Sympathy is sure to be extended to Dr. Balfour for the severe loss the laboratories sustained by the fire of May 11. The office files for five years, books, and sanitary records all perished, besides equipment, cultures, and other things. A boat fitted up as a floating laboratory has proved a great success; so far towed from place to place by the post boats, Mr. King thinks it would be more useful if it had its own launch attached. This certainly would be the case, as then it would be quite independent and could move anywhere as required. Dr. Wenyon worked in this and collected much of his material when in charge of it up the Nile. Out of the many different papers in the report some will prove of greater interest than others to individuals, this largely depending on their own scientific taste. The one on economic entomology deserves special mention as being of great practical utility. Insect pests are the bugbear of fruit-growers and cultivators of the soil, just as bacteria and protozoa are of the physician. The coloured plates in this section, though good in their way, are not up to the standard of the other illustrations, and the work of the artist Terzi, seen in previous reports, is sadly missed. Amongst purely medical matters it is disquieting to note that kala azar—that deadly Indian disease—is present and apparently not rare in the Soudan. As regards the diagnosis of this complaint, it might be suggested to Captain Bousfield that puncturing the spleen is not so devoid of risk as he thinks. Deaths have occurred from the operation and are certain to do so again; so, as the diagnosis can be made quite as easily from liver-blood, this is a preferable method. It is interesting to note (vide Captain Cummin's paper) that the parasites were never found in the leucocytes in the peripheral blood of any of the cases in the Soudan. Cases from India studied in England have shown a similar peculiarity, and though Patton (in India) has often found them in some of his cases it is not altogether clear how the bug is always to be certain of getting the infection (if that insect does eventually prove to be the intermediate host). Another interesting medical point (mentioned on p. 114 of Captain Bousfield's paper) crops up in the following statement: "The presence of albumin in the urine is important, for in the severer cases of 2,000 odd malaria patients last autumn albumen was never found except where something else was present to account for it, such as bilharziosis." Certainly in the malignant fevers seen in many parts of the tropics albumin in considerable quantity is quite a common occurrence; so much so is this the case in the West Indies that it considerably hampers the diagnosis of the early cases of yellow fever. A point such as this is well worth working at; it might even indicate that there are different varieties or species of the malignant parasite, or at least brands of different virulence. The purely scientific papers are of a high standard, but perhaps are a little too advanced for the ordinary medical individual; for example, Dr. Wenyon's criticism of Schaudinn's work on the leucocytozoa and other finer points in protozoology are only of use to the experts. Tropical medicine is still a rich gold mine for the research worker, and results come so quickly that one cannot wonder at the large number of men devoting their time now to this branch of medicine. A very good report containing work and illustrations of high excellence sums up the publication in a nutshell.

### THERAPEUTICS.

A Manual of Medical Treatment, or Clinical Therapeutics. By I. Burney Yeo, M.D., F.R.C.P., Emeritus Professor of Medicine in King's College, London, etc.; Raymond Crawfurd, M.A., M.D., F.R.C.P., Physician to King's College Hospital; and E. Farquhar Buzzard, M.A., M.D., F.R.C.P., Assistant Physician to the Royal Free Hospital. Twenty-second Thousand. New edition, in two volumes. (Cassell and Co., Ltd. 1909. Pp. 802 and 829, and 29 figs. Price, 21s. net.)

This is the fourth edition of a work which is so well known that little need be said about it. The third edition appeared in 1902, since which time there has been a remarkable development in therapeusis, particularly along the lines of vaccine treatment. The authors are very cautious in their views upon the value of the latter, so that the reader has the advantage of a certain amount of scepticism in the descriptions given. There is an index extending over 42 pages, arranged chiefly under clinical headings; we think it a pity, perhaps, that medicinal headings are not given also, notwithstanding that the index is already of great length. The various conditions under which different mineral waters are employed require more than five columns, a degree of detail in an index that is admirable: but should one wish to look up the mode of administration of musk, or atoxyl, or lactobacillin, and so forth, one cannot find them in the index at all. Notwithstanding minor points such as these, the work as a whole is worthy of the highest praise. Dr. Burney Yeo has been ably assisted, both by Dr. Crawfurd and by Dr. Buzzard. The latter has been responsible for the revision of the part treating of diseases of the nervous system; the former for similar revision of the chapters upon digestive and circulatory diseases, and those dealing with maladies of the liver and kidneys. The work is one which every practitioner will find of the greatest practical service.

Physical Methods in the Treatment of Heart Disease. By Arthur G. Dampier-Bennett, M.R.C.S. (Bristol: Jno. Wright and Co. 1907. Pp. 111. Price 3s. 6d. net.)

This is a very practical and well-written manual. The author has evidently studied his subject in a very thorough fashion, and his conclusions and comments are the outcome of a careful and exact experience. Physical methods of treatment have undoubtedly in recent years commanded more attention from the profession than was at one time the case, but even yet they hardly receive the consideration they deserve. The book now before us ought to help to remedy this defect, and we can heartily commend it to the practitioner who is anxious to deal effectively with cases of chronic heart disease. The several chapters of the volume include the uses of the Nauheim and other baths, various forms of massage, active and passive exercises, electrical applications, and diet. Some of the most valuable sections are those dealing with the wise selection of cases for the various methods discussed. The book is written in a clear and lucid style and is marked throughout by a dignified and scientific spirit.

### SURGERY.

Injuries and Diseases of the Knee-joint. By Sir Wm. Bennett, K.C.V.O., F.R.C.S. (London : James Nisbet and Co., Ltd. 1909. Pp. 236, 34 illustrations. Price 5s. net.)

Sir William Bennett, in his new book, confines himself very successfully within certain limits which he has marked out for himself. His treatise is essentially clinical, and it embodies a series of essays which form really admirable post-graduate lectures, and were many of them actually delivered as such. Operative technique and the details of antiseptic methods, for instance, are omitted altogether, the latter, no doubt, as too elementary, the former possibly as requiring detailed consideration as in the more ambitious works on operative surgery. Again, a passable acquaintance with the anatomy of the knee is assumed, and with the details of ordinary forms of mechanical treatment such as splinting, bandaging, etc. This standpoint makes possible within a reasonable compass and at a moderate price a collection of sound and practical advice about conditions of everyday occurrence which are of most vital concern to the general practitioner.

The book is far from giving a complete synopsis of the very large subject which is comprehended within its title, as the author very candidly admits at the outset. But it does deal efficiently with the clinical side of whatever lesion is being discussed, and especially it includes many hints and practical suggestions on diagnosis and treatment not as yet to be found in the systematic treatises on surgery. A full account is given of the peculiar synovial distensions which Sir Wm. Bennett has been the first to describe, unless we err, in connection with puberty, menopause, or menstrual derangement; and the chapters on displaced cartilage and internal derangement of the knee-joint are adequate to the importance of the subject. The differential diagnosis of many of the most difficult diseases and injuries are illustrated by well selected and succinctly recounted clinical cases from the author's wide experience, and many of these are of very great interest. To attempt to convey the pith of Sir Wm. Bennett's teaching by quotation here would be useless; rather do we recommend all surgeons and general practitioners to read the volume through for themselves. The want of an index is a drawback which seems a great pity, as a very modest one would suffice, and would much enhance the value of the book.

Seven Hundred Surgical Suggestions : Practical Brevities in Diagnosis and Treatment. By Walter M. Brickner, Eli Moschowitz, and Harold M. Hays, the Editors of the *American Journal of Surgery*. (New York : Surgery Publishing Company, 92 William Street, New York, U.S.A. 1909. Pp. 150 + iii.)

This book consists, as its name implies, of a number of surgical aphorisms of greater or less importance, which have occurred to its authors from time to time as being valuable from a practical point of view. The observations vary in utility; some of them are excellent, many are sound, and a few are superfluous or misleading. As an instance of the last we may quote the following : "A very simple method of curing a corn is to excise it." Now, in our experience a corn will always recur, whatever surgical measures have been adopted, as long as its site is still subjected to pressure. With some of the statements expressed we venture to disagree; for instance, that "in many, if not most, cases of tuberculous testis the seminal vesicle or prostate is involved, and it will be necessary to remove one or other of these structures in order to cure." It is true that these structures are often involved, but the operation for removing them is a heroic one. It is better to wait until an abscess has formed and then to evacuate it. The question of the utility of publishing such a book is debatable. Of its use to the authors there can be no question. Such a book ought to be kept by everyone engaged in the practice of surgery. It might justly be called "a book of useful knowledge." It will serve to remind him of his omissions and mistakes, and should prevent him from making them again. But it can never be of as much value to the world at large as it is to him who collected it; and that is the sum of our criticism of this work. The style is good, and we have nothing but admiration for the clearness of its arrangement.

### ZOOLOGY.

The Origin of Vertebrates. By W. H. Gaskell, M.A., M.D., F.R.S. (London : Longmans, Green and Co. 1908. Price 21s.)

With Dr. Walter Gaskell's reputation as a physiologist every medical man whose physiological studies have been pursued during the last quarter of a century is familiar. His best-known researches in this field are probably those connected with the structure of the heart and the modes of origin and control of the cardiac beat; researches which have only quite recently been extended to the problems of human heart-disease by various investigators in this country and abroad. Not satisfied with the achievement of high distinction in physiology, Dr. Gaskell has for many years applied himself to one of the most knotty problems of the sister-science of zoology—that is, the manner of origin of the vertebrates from the invertebrate kingdom. Throughout his physiological teaching, as those who have been fortunate enough to attend his Cambridge lectures know, he has found time to impress his classes with the fascinating study of developmental processes, and has contributed much to biological literature on this problem which has more especially engaged his attention. He now summarises in book form the conclusions he has arrived at and the chain of argument which has led him to them. Put very briefly, Dr. Gaskell believes that he can trace the vertebrate ancestry to the group known as the *Palæostraci*, of whom the sole living descendant is the king-crab, a peculiar animal which is sometimes displayed as a curiosity in fishmongers' windows. This group is believed to have arisen in Silurian and Devonian times from the great tribe of trilobites; its members were in their day the predominant race on the earth, existing in multitudes of species and countless numbers. From the *Palæostraci* may have been derived both the crustaceans and the arachnids, which in that case must both be regarded as side tracks from the main line of vertebral descent. Into the extraordinarily ingenious arguments with which this hypothesis is supported we cannot now enter; every system is ransacked in turn for confirmatory evidence, and the most unlikely clues are turned to advantage. Thus the thyroid gland is pronounced to represent the uterus of the invertebrate; this is put forward on the strength of embryological as well as anatomical data, and it is even suggested that it may be the explanation of a certain subtle connection between this gland and the sexual organs of the vertebrates, including man. That, however, is somewhat by the way; the pivot on which the whole construction hinges is the identification of the neurenteric canal of the vertebrate with the primitive gut of the invertebrate, which lies, it may be remembered, dorsal to all the segmental ganglia except the first two. The infundibulum represents this original alimentary canal where it passed between the rami connecting the præ-œsophageal with the post-œsophageal ganglia, and the ventricles of the brain represent the same canal where the enveloping mass of nervous tissue has failed completely to roof it over. Enough has been said to show that Dr. Gaskell is nothing if not resourceful; for the full enjoyment of his interesting hypothesis we must refer readers to his treatise, though they should be warned that it is rather stiff reading, in the same sense as is any other biological book worth studying. That is to say, it requires close attention in the reader to the logical sequence of the argument. We think the professional zoologists do not agree with him on the question of the notochord, which is dismissed as unworthy of the prominence which has been bestowed upon it; and indeed there are no doubt many other points on which they will express dissent. However, Dr. Gaskell's arguments have to be met; and even if that comparatively easy form of energy, destructive criticism, should shake some of his conclusions, the scientific world will still be in his debt for a very courageous and notable addition to literature.

### MISCELLANEOUS ITEMS.

The Local Government Annual. Price 1s. 6d. Departmental Decisions by the Local Government Board, Home Office, Treasury, etc. No 13. Price 2s. (London: *The Local Government Journal* Office. 1909.)

With the extension of the Public Health and other preventive services there are increasing numbers of the medical profession who become annually connected in various capacities with some or other part of the machinery of Local Government. To any who are thus employed, and to all who are interested in the subject by active participation in the work of Local Government on municipal or urban district or county councils, this annual is a work of reference which can be confidently recommended. Hardly less valuable to medical officers of health and other officials of the public medical services is the collection of departmental decisions of various Government offices contained in the second of the two booklets named above, which includes several important official pronouncements bearing directly on the duties and privileges of medical men in certain of their public capacities. There are added also a number of legal decisions on important points; these do not directly bear on medical practice, but many of them are extremely interesting to everyone in his capacity as a private citizen.

An Introduction to the Science of Radio-Activity. By C. W. Raffety. 200 pages, with illustrations. (Published by Longmans, London. 4s. 6d. net.)

There is perhaps no more difficult task in the range of popular science than the explanation of the results which have been obtained by very advanced mathematical calculation. For this reason the advice of Faraday, that the writer should assume complete ignorance on the part of his readers, is likely to produce the best effects. Mr. Raffety divides his book very lucidly into three parts: the first of which is descriptive, the second theoretical, and the third practical. He discusses the radio-active effects of uranium, radium, thorium, and actinium in some detail, and certainly leaves on the mind of the reader a clear idea of the significance of $\alpha$ $\beta$ and $\gamma$ rays. We suggest, however, that a footnote explaining the meaning of the word "kathode" is a great desideratum if he wishes to appeal to those whose acquaintance with electricity is small. In fact, excellent as the book is in many ways, it leaves a feeling of incompleteness, over and above the deficiency in this respect which can be explained by the brevity of treatment. It is not often that we wish for more words, but in Mr. Raffety's case the language is concise even to obscurity. There are, too, occasional lapses from the rules of English composition. The illustrations are instructive and well reproduced. We think, however, that if Mr. Raffety had devoted 400 pages to his work, the book would have avoided all the faults we mention. The theoretical part, containing roughly 60 pages, might have been amplified to great advantage. The appendix is most useful.

The Food Inspector's Handbook. By Francis Vacher, Medical Officer of Health for Cheshire. Fifth Edition. (London: The Sanitary Publishing Co., Ltd. 1909. Pp. xxiv. + 268. Price 7s. 6d. net.)

Nearly four years have passed since the publication of the fourth edition of this well-known handbook. The new edition is much enlarged, and the section on meat—which is, of course, one of the most important parts of the book—has been virtually rewritten. Other additions include the text of the Butter and Margarine Act, 1907, and of the recent regulations of the Local Government Board on unsound food and foreign meat respectively. The handbook is intended primarily to be in the nature of a practical guide for medical officers of health and sanitary inspectors. The treatment of some of the questions discussed appears to lack thoroughness, but that fact is inevitable in a pocket "handbook," and the author would doubtless be the first to admit that he has not attempted to deal exhaustively with his subject-matter. The treatment of the Sale of Food and Drugs Act is a little unsatisfactory. A paraphrased version of the text of the Act of 1875, with so little explanatory comment, is valueless to persons who are engaged in administration. The illustrations show a substantial improvement as compared with those in previous editions.

The Body at Work. By Alex Hill, M.A., M.D., F.R.C.S. 440 pages. 46 illustrations. (Published by Arnold, London. 16s. net.)

Professor Huxley used to maintain that the demand upon a writer who wishes to write popularly is as great as upon the man whose audience is more circumscribed. The chief obstacle in the way of success is the extreme difficulty of deciding how much the amateur of science may be presumed to know. The great sin in all popular scientific writing is sensationalism, and the avoidance of it very frequently produces obscurity for the lay reader. Dr. Hill has certainly attained considerable success in this effort of his to deal with the fundamental problems of physiology. He begins with the simple consideration of the cell, and thence passes to a review of the various functional parts of the body which is composed of cells. In dealing with bacteria and the work of the leucocytes, Dr. Hill seems to write very happily, but we fear that the chemical aspect of digestion presupposes a greater knowledge of chemistry than is possessed by the ordinary scientific amateur. For the most part the language is well chosen and the sentences run smoothly. We welcome certainly this recognition of the philosophy of physiology, and we are inclined to think that this side of the subject is too frequently omitted even in more technical works. The illustrations, if not striking, are adequate, and the index is most acceptable.

